# Prognostic Nomogram for Prediction of Axillary Pathologic Complete Response After Neoadjuvant Chemotherapy in Cytologically Proven Node-Positive Breast Cancer

**DOI:** 10.1097/MD.0000000000001720

**Published:** 2015-10-30

**Authors:** Jee Ye Kim, Hyung Seok Park, Sanghwa Kim, Jegyu Ryu, Seho Park, Seung Il Kim

**Affiliations:** From the Department of Surgery, Yonsei University College of Medicine, Seoul, Korea (JYK, HSP, SK, JR, SP, SIK).

## Abstract

To develop a nomogram predicting probability of axillary pathologic complete response (pCR) in patients with cytologically proven axillary node-positive breast cancer who received neoadjuvant chemotherapy (NAC).

The current management of axillary intervention in node-positive breast cancer patients who received NAC is axillary lymph node dissection (ALND) regardless of axillary pCR.

We reviewed the records of 415 patients with cytologically proven node-positive breast cancer that were treated with NAC followed by surgery between 2008 and 2012 at Severance Hospital, Yonsei University Health System. Baseline patient and tumor characteristics, chemotherapy regimen, and tumor and nodal responses were analyzed. A nomogram was developed using a binary logistic regression model with a training cohort and validated in an independent cohort of 110 patients.

Axillary pCR was achieved in 38.8% of the patients who underwent ALND after NAC. Axillary pCR was associated with initial clinical nodal status, negative estrogen receptor status, positive human epidermal growth factor receptor 2 (HER2) status with trastuzumab, and clinical nodal and tumor responses. A nomogram was developed based on the clinical and statistically significant predictors. It had good discrimination performance (AUC 0.82, 95% CI, 0.78–0.86) and calibration fit. The nomogram was independently validated, indicating the good predictive power of the model (AUC 0.80, 95% CI, 0.72–0.88).

Our nomogram might help predict axillary pCR after NAC in patients with initially node-positive breast cancer. Patients with a high probability of achieving axillary pCR could be spared ALND, avoiding postoperative morbidity.

## INTRODUCTION

Neoadjuvant chemotherapy (NAC) has become a widespread treatment modality in locally advanced breast cancer.^1,2^ It can safely downstage operable breast tumors so that conservation surgery can be used as an alternative to mastectomy.^3^ Furthermore, the neoadjuvant setting offers the opportunity to assess the response to NAC by an in vivo chemosensitivity test.

NAC results in the negative conversion of a metastatic axillary lymph node in 22% to 41%^4–8^ of patients with cytologically confirmed node-positive breast cancer. An axillary pathologic complete response (pCR) is associated with excellent prognosis,^4,8^ and patients who achieve axillary pCR can be spared axillary lymph node dissection (ALND), avoiding postoperative morbidities such as lymphedema, arm pain, and reduced arm movement.^9,10^ Until now, axillary pCR has could only be determined by performing ALND.

The current standard for axillary staging in clinically node-negative patients is sentinel lymph node biopsy (SLNB).^11^ ALND is recommended for axillary intervention in patients with clinically node-positive breast cancer who received NAC,^11^ because the feasibility of SLNB is uncertain in those patients.^6,7^ Recent prospective multicenter trials found that SLNB following NAC had a low detection rate (80.1–92.7%) and a relatively high false-negative rate (12.6–14.2%).^5,12^ SLNB could be available, however, with more or less a 90% detection rate and a 10% false-negative rate under certain conditions, including the detection of 2 or more SLNs and the use of dual mapping. Those findings suggest that along with SLNB, additional tools might be helpful to evaluate the axillary lymph node response to NAC in patients with cytologically proven node-positive breast cancer and to identify patients in whom ALND could be omitted.

A nomogram in a clinical setting is considered a comprehensive predictive tool that can estimate the probabilities or risks or clinical outcomes. In previous studies, nomograms provided detailed probabilities or risks of clinical outcomes for clinicians and patients making decisions in the management of breast cancer.^13,14^ There are few well-designed nomograms predicting the probability of axillary pCR in the literature, and the implications of providing a detailed probability of axillary pCR in patients who receive NAC are well not established.

For those reasons, we investigated the factors that predicted axillary pCR and established a nomogram calculating the probability of axillary pCR in patients with cytologically proven axillary node-positive breast cancer who received NAC.

## PATIENTS AND METHODS

### Study Population

Patients with clinical T1–T4, N1–N3, and M0 primary invasive breast cancer who received NAC followed by curative surgery between January 2008 and July 2014 at Severance Hospital (Seoul, South Korea) were retrospectively reviewed. Among 662 patients, 2 male patients, 120 patients without cytologically proven axillary lymph node metastasis, and 8 patients who underwent ALND only were excluded. All the 525 patients had node-positive disease confirmed by fine-needle aspiration biopsy at presentation and underwent radical operation of the primary tumor with concurrent ALND.

**FIGURE 1:**
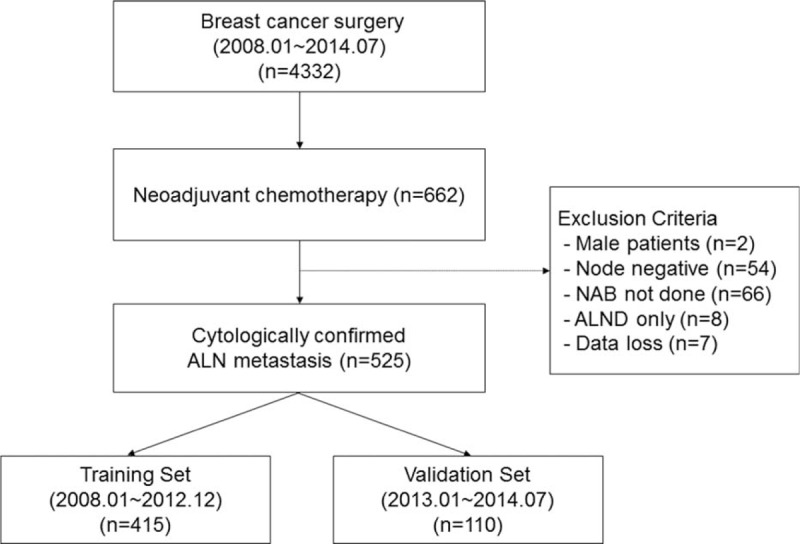
Flowchart of patient selection. NAB = needle aspiration biopsy, ALND = axillary lymph node dissection.

Among 525 patients, 415 patients treated between January 2008 and December 2012 were evaluated and used as a training set to develop a nomogram. The nomogram was validated independently using a set of 110 patients. All the patients in the validation set also had cytologically confirmed node-positive disease and received NAC followed by curative surgery between January 2013 and July 2014 in the same institution (Fig. 1). This study was reviewed and approved by the Institutional Review Board of Severance Hospital, Yonsei University Health System (2014-2789-001).

### Clinicopathological Characteristics and Evaluation of Responses to NAC

Patients were divided into 2 categories of age 35 and below, versus above age 35.^15,16^ The body mass index (BMI) was calculated on the date of the first course of NAC. BMI ≥25 kg/m^2^ was classified to obesity according to Asia-Pacific Region Criteria of WHO classification.^17^

Before NAC, estrogen receptor (ER), progesterone receptor (PR), human epidermal growth factor receptor 2 (HER2), Ki67 expression, and histologic grade of the diagnostic core biopsy sample were evaluated. Tumors with ≥10% nuclear-stained cells were considered positive for ER and PR. Tumors with HER2 3+ overexpression determined by immunohistochemistry or HER2 amplification determined by in situ fluorescence were considered to overexpress HER2.^18^ Ki67 expression ≥14% was considered high.^19^ Histologic grade was assessed using the modified Bloom–Richardson classification.^20^ The clinical and pathologic stage was assessed according to the 7th edition of the AJCC cancer staging system.^21^ Clinical N3 was defined by physical examination, cytological confirmation, and imaging including MRI and PET-CT.

The biologic subtypes were categorized into 4 subgroups according to ER, PR, and HER2 status as follows: luminal A, ER positive and/or PR positive, and HER2 negative; luminal B, ER positive and/or PR positive, and HER2 positive; HER2-enriched, ER negative and PR negative and HER2 positive; triple-negative breast cancer, ER negative and PR negative and HER2 negative.

Chemotherapy regimens were classified as anthracycline and taxane, anthracycline and no taxane, or no anthracycline and no taxane.

The clinical tumor size was measured as the largest single tumor diameter on ultrasound before and after the completion of NAC. Malignant microcalcification was determined by the presence of intratumoral microcalcification on ultrasound. Multifocality/multicentricity was defined by the presence of biopsy-proven multifocal and/or multicentric tumors in the ipsilateral breast.

The clinical tumor response rate to the NAC was calculated by the rate of tumor size reduction, comparing the largest single tumor diameter on the ultrasound image at baseline to the largest single tumor diameter on the ultrasound image after NAC ([icT-ycT]/icT). Classification according to the Response Evaluation Criteria in Solid Tumors ver. 1.1^22^ was also utilized. A complete response was defined as the complete or near-complete resolution of the lesion. A partial response was defined as a 30% or greater decrease in the size of the lesion. Disease progression was defined as a 20% or greater increase in the size of the lesion. All other responses were defined as stable disease. The clinical nodal response was determined by ultrasound description of the presence or absence of suspicious and malignant-appearing lymph nodes after the completion of NAC.

All the patients underwent breast and axillary surgery within 6 weeks of completing NAC. The type of breast surgery was selected according to the preferences of the surgeon and the patient. ALND was performed for axillary nodal metastasis regardless of accompanying SLNB. Patients with cytologically proven supraclavicular and/or infraclavicular lymph nodes were treated with concurrent neck node dissection. The pathologic response was evaluated using the largest single diameter of the invasive tumor in the surgical specimen. Axillary pCR was defined as the absence of macrometastasis and micrometastasis.

### Model Construction and Performance Evaluation

We built a nomogram based on a binary logistic regression model with the significant and predefined predictors in the training cohort. The model was then used to predict the probabilities of individual patients achieving axillary pCR to NAC.

The performance of the model was quantified with respect to discrimination and calibration. Discrimination is the predictor's ability to separate patients with different responses or events. The discriminatory abilities of the model were assessed by measuring the area under the receiver-operating characteristics (ROC) curve. Calibration is the agreement between the frequencies of observed outcomes and the probabilities predicted by the model. The calibration plot was evaluated using the Hosmer–Lemeshow goodness-of-fit test and visualized by plots.

### Statistical Analysis

Relationships between variables were studied using standard tests. Continuous variables were compared using 2-sample *t* tests, and categorical variables were compared between and among groups using a Chi-square test or a Fisher exact test. Univariate and multivariate binary logistic regression analyses were used to identify factors associated with axillary pCR after NAC. Odds ratios and 95% confidence intervals were calculated. All statistical tests were 2-sided, and *P*-values less than 0.05 were considered statistically significant.

Analyses were performed using Statistical Program for Social Science (SPS) version 20.0 (IBM, Inc., Chicago, IL) and R Statistical Package (Institute for Statistics and Mathematics, Vienna, Austria, Ver. 2.9.2 and Ver. 3.1.1, www.R-project.org).

## RESULTS

### Patient Characteristics

The baseline patient and tumor characteristics for the 415 patients in the training set are shown in Table 1. Axillary pCR was achieved in the 38.3% of the patients. Patients with axillary pCR tended to be younger (≤35 years), and were more likely to have tumors with early clinical and nodal stage compared with patients with axillary non-pCR. Tumors with high histologic grade, negative ER and PR status, positive HER2 status with trastuzumab, and high Ki67 expression were significantly more common in patients with axillary pCR. BMI, histologic type, and malignant microcalcification were not significantly different between the patients with and without axillary pCR, respectively. There was a trend for a higher percentage of triple-negative (31.4%) and HER2-enriched breast cancers (23.9%) among the patients with axillary pCR compared with those with axillary non-pCR (16.8% and 15.2%, respectively; *P* < 0.001).

**TABLE 1:**
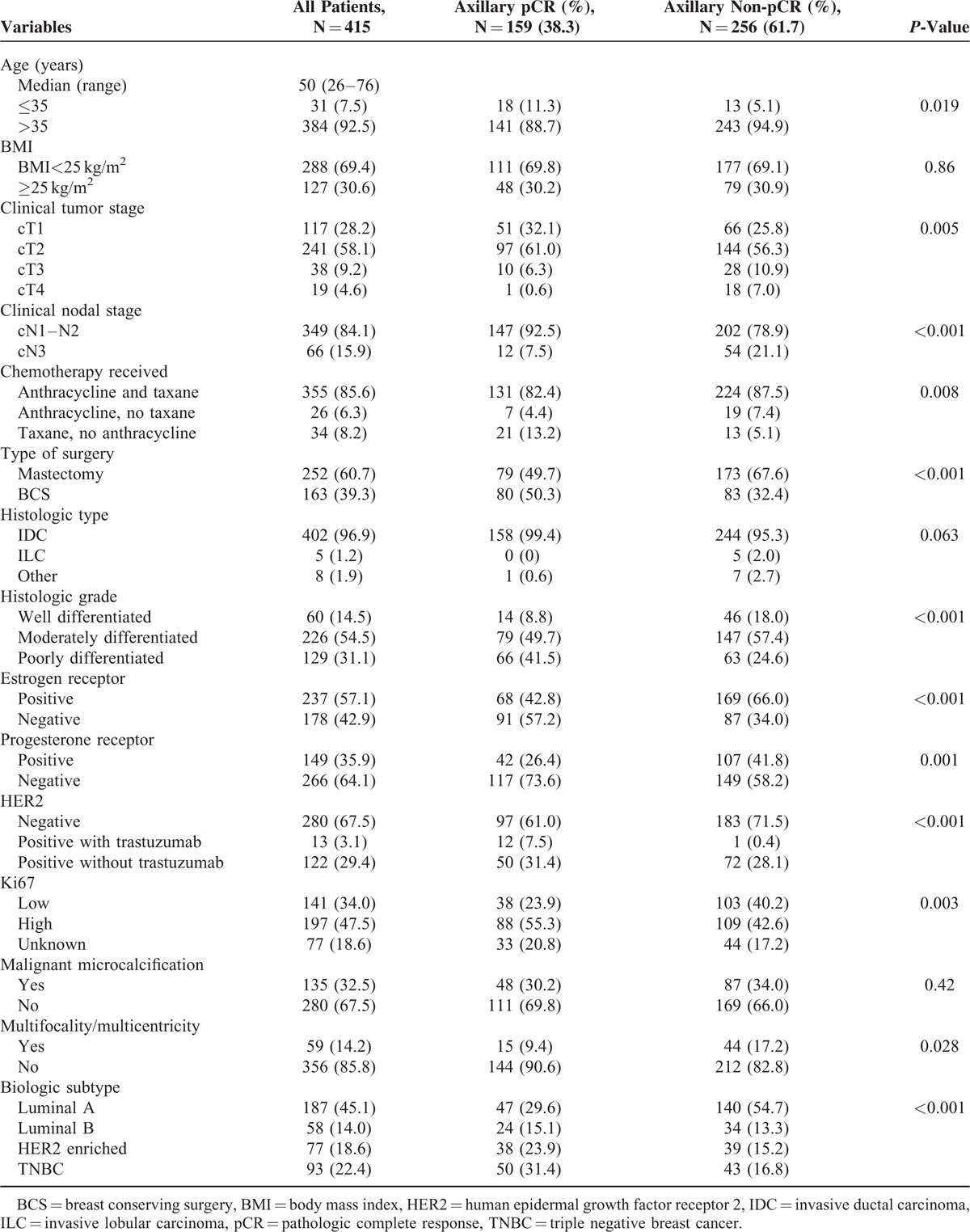
Baseline Patient and Tumor Characteristics of 415 Patients Treated With Neoadjuvant Chemotherapy

The baseline patient and tumor characteristics for 110 patients in the validation set are shown in Supplemental Table 1.

### Clinicopathologic Response to Neoadjuvant Chemotherapy

The median clinical tumor diameters before and after NAC were 3.2 cm (range, 0.2–10.0 cm) and 1.6 cm (range, 0–10.0 cm), respectively (Table 2). The clinical tumor size throughout the treatment was significantly smaller in the patients with axillary pCR. The clinical response rate of the breast tumor was 51% overall and was significantly higher in the patients with axillary pCR compared with that in the patients with axillary non-pCR (67% and 42%, respectively; *P* < 0.001). The rate of clinical complete response of the breast tumor was significantly higher in the patients with axillary pCR compared with that in the patients with axillary non-pCR (24.5% and 8.2%, respectively; *P* < 0.001).

**TABLE 2:**
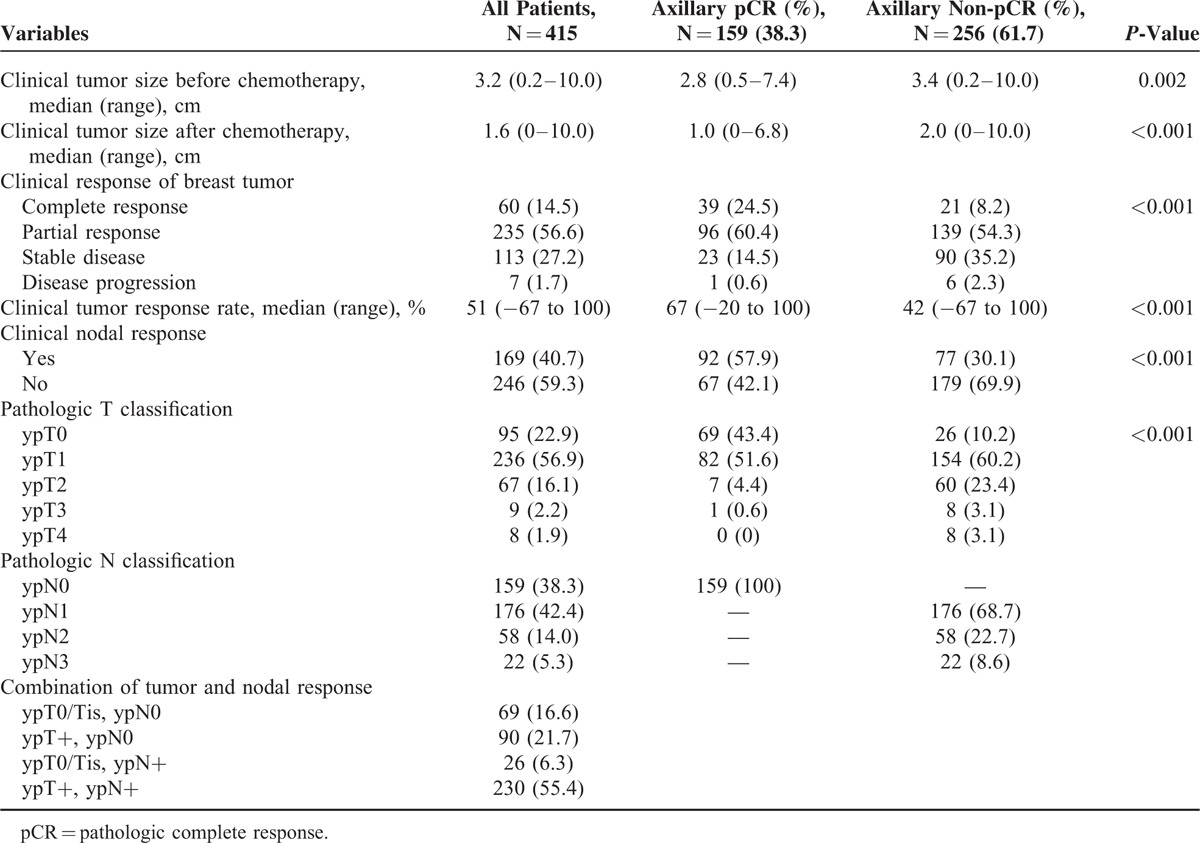
Clinical and Pathologic Response to Neoadjuvant Chemotherapy

The rate of tumor pCR (ypT0/Tis) was significantly higher in the patients with axillary pCR compared with that in the patients with axillary non-pCR (43.4% and 10.2%, respectively; *P* < 0.001). Among the patients who did not achieve axillary pCR, 68.8% had ypN1 disease, 22.7% had ypN2 disease, and 8.6% had ypT3 disease. The overall rate of pCR in both the breast and the axilla (ypT0/Tis, ypN0) was 16.6%.

Clinicopathologic response to NAC of the patients in the validation set is shown in Supplemental Table 2.

### Significant Predictors of Axillary pCR

The univariate logistic regression analysis of the 415 patients in the training set who underwent ALND after NAC showed that the proportion of patients with axillary pCR increased with high histologic grade, young age, early clinical tumor stage, and nodal stage. Breast conserving surgery, taxane-based chemotherapy, negative ER status, negative PR status, positive HER2 status (especially with trastuzumab treatment), high Ki67 expression, and absence of multifocality/multicentricity were associated with axillary pCR as the clinical tumor or clinical nodal response.

The multivariate logistic regression analysis showed that early clinical nodal stage, negative ER status, positive HER2 status with trastuzumab treatment, and clinical tumor and nodal response were significantly correlated with an increased probability of achieving axillary pCR (Table 3).

**TABLE 3:**
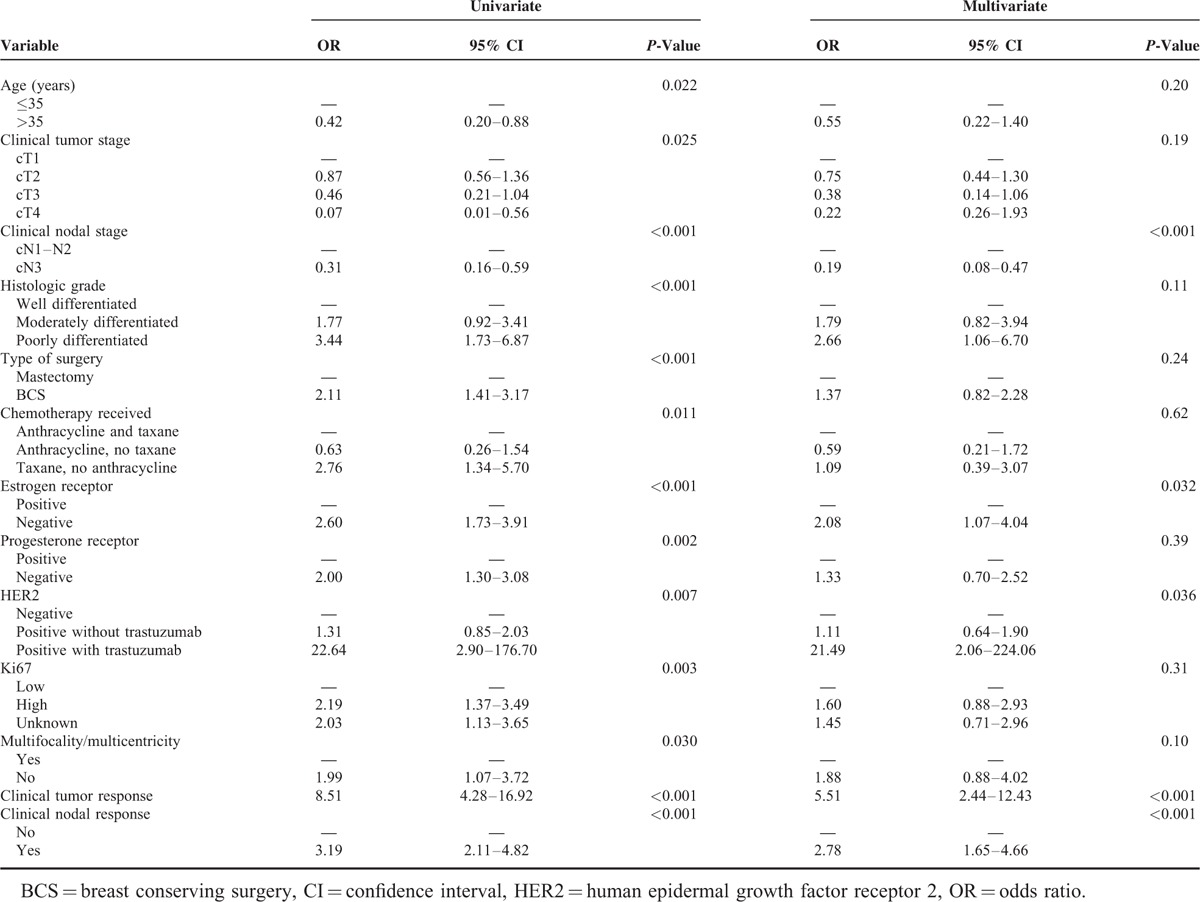
Univariate and Multivariate Analysis for Axillary Pathologic Complete Response (n = 415)

### Development and Validation of the Nomogram

We constructed a nomogram based on the clinically and statistically significant variables. The significant variables that can be assessed in preoperative evaluations in the univariate analysis were years of age (≤35 vs. >35), clinical tumor stage (cT1, cT2, cT3, or cT4), histologic grade (well, moderate, or poor), Ki67 (low vs. high expression), and regimen of NAC (anthracycline and taxane, anthracycline and no taxane, or taxane and no anthracycline).

**FIGURE 2:**
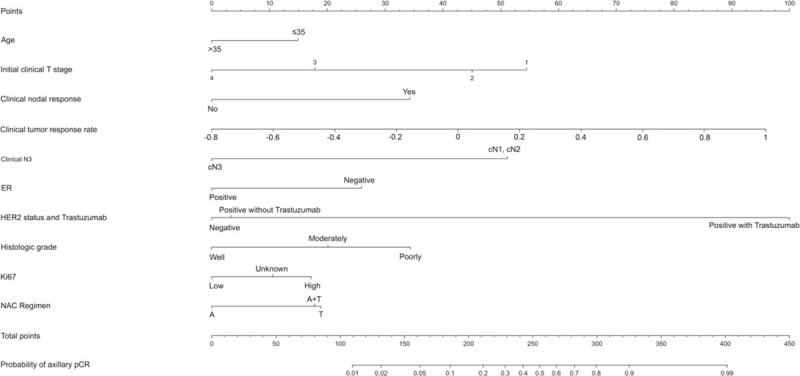
Nomogram for prediction of the probability of axillary pathologic complete response. NAC = neoadjuvant chemotherapy, A = anthracycline, T = taxane, pCR = pathologic complete response.

Fig. 2 illustrates the nomogram to calculate the probability of achieving axillary pCR. The total nomogram score is calculated by summing up the scores for each of the variables. The total score can then be used to assign a probability of achieving axillary pCR to individual patients using the scale at the bottom of the figure.

**FIGURE 3:**
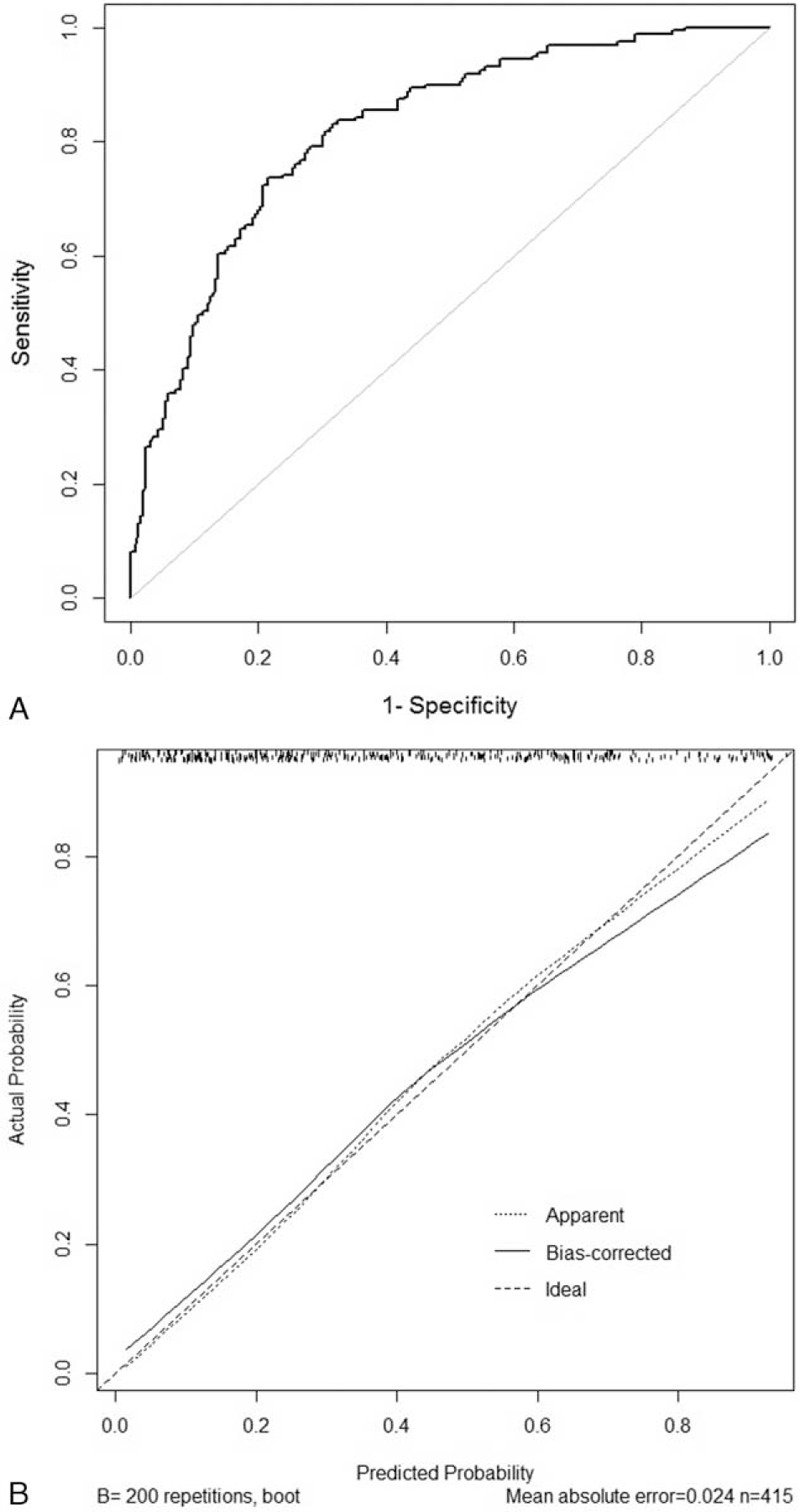
The receiver-operating characteristics (ROC) curve and the calibration plot of the nomogram in the training set. (A) ROC curve with AUC = 0.82 (95% CI, 0.78–0.86). (B) Calibration plot of the nomogram (Hosmer–Lemeshow test: *P*-value = 0.94).

The ROC curve of the nomogram is shown in Figure 3A. The area under the ROC curve was 0.82 (95% CI, 0.78–0.86), indicating the good predictive power of the model. The calibration plot (Fig. 3B) showed good agreement between the predicted and observed probabilities according to a Hosmer–Lemeshow test (*P*-value = 0.94).

**FIGURE 4:**
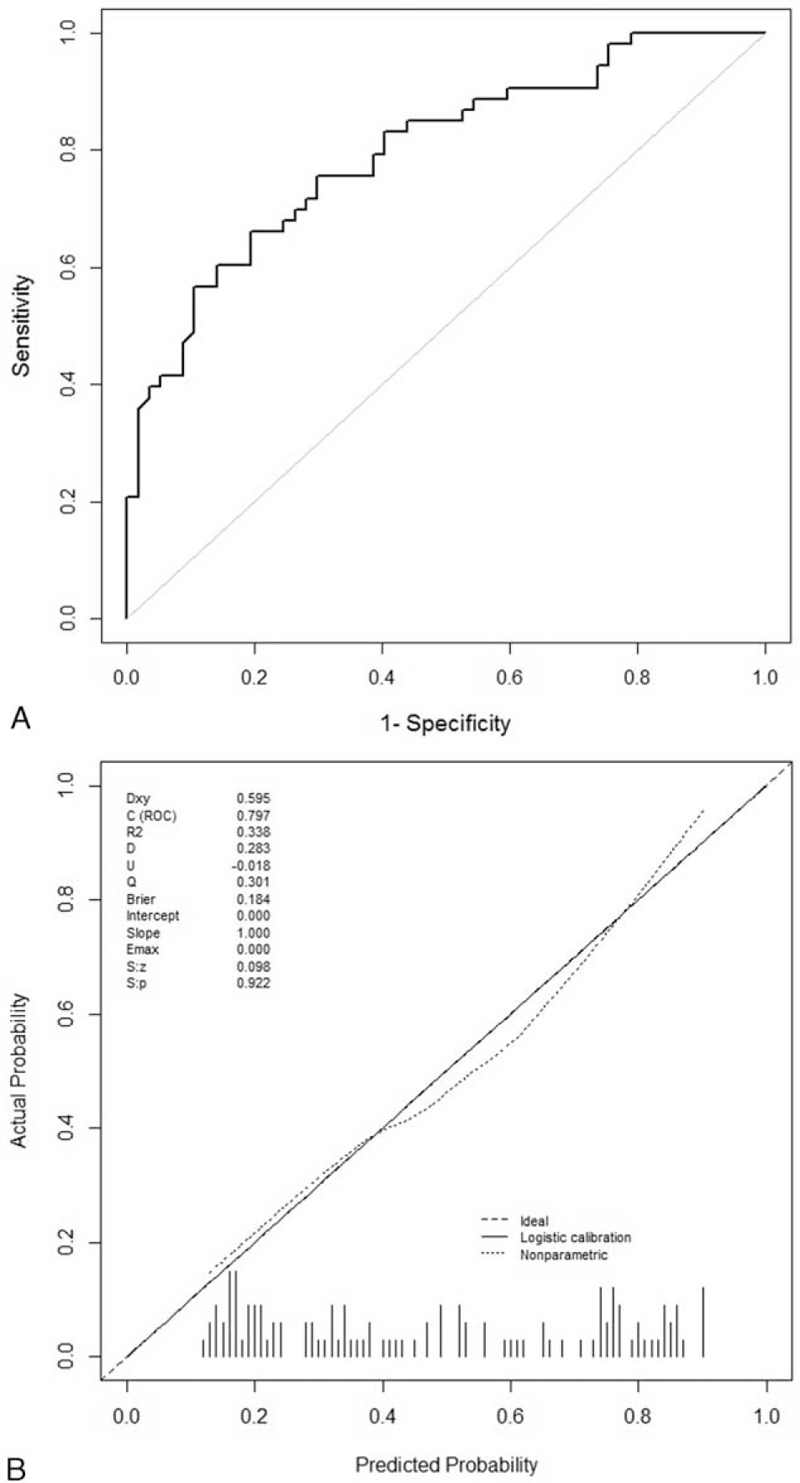
The receiver-operating characteristics (ROC) curve and the calibration plot of the nomogram in the validation set. (A) ROC curve with AUC = 0.80 (95% CI, 0.72–0.88). (B) Calibration plot of the nomogram (Hosmer–Lemeshow test: *P*-value = 0.19).

The model was applied to an independent cohort of 110 patients who met the eligibility criteria for validation. Figure 4A shows the ROC curve for the validation set. The area under the ROC curve was 0.80 (95% CI, 0.72–0.88), indicating the substantial predictive power of the model in the validation set. The calibration plot (Fig. 4B) yielded good agreement between the expected and actual events in the validation set. Hosmer–Lemeshow test of goodness-of-fit was not significant (*P*-value = 0.19), indicating the good fit of the model.

## DISCUSSION

Predicting pCR of the axillary lymph node after NAC in patients with breast cancer and cytologically confirmed nodal metastasis is important for understanding patient outcomes as well as for identifying patients in whom ALND might be omitted. We performed a retrospective review of patient data and developed a prediction model based on the clinicopathologic characteristics of the primary tumor to estimate the probability of achieving axillary pCR. We then validated the model in an independent cohort of patients.

In this study, axillary pCR was achieved in 38.3% (159 of 415) of the patients, which is similar to the rates seen in previous studies.^4–7^ It has been shown that tumors that are ER-negative,^23,24^ HER2-positive with trastuzumab treatment,^25,26^ have a high histologic grade,^27^ or have high levels of Ki67 expression^28^ are more likely to achieve pCR. As anticipated, patients achieving axillary pCR not only had lower disease burden of the primary tumor and the lymph node but also had a higher percentage of tumors that achieved pCR. The clinical tumor response rate and the nodal response to NAC were also highly associated with axillary pCR.

Our nomogram is in line with previous studies predicting axillary pCR after NAC in patients with cytologically proven nodal metastasis,^4,29^ but it has several advantages compared with previous models. Although the previous models had acceptable performance with standard variables, they lacked proliferation scores and did not consider tumor and nodal responses to the NAC. In addition, they were not validated in a separate sample but internally validated by bootstrapping.

We developed a nomogram with improved performance by adding proliferation markers such as histologic grade and Ki67 expression. Histologic grade and Ki67 expression level have been investigated previously as predictive markers for responses to NAC,^28,30^ so we incorporated them into the nomogram after finding that they were associated with axillary pCR in our univariate analysis. Another advantage of the nomogram is that the clinical responses of the primary tumor and the axillary lymph node to NAC were considered important factors for the prediction of axillary pCR. Ultrasound is included in the standard workup procedures around the NAC in our institution, because the primary tumor response can be easily obtained by measuring the tumor diameter by ultrasound before and after NAC. The lymph node response can also be reflected by the presence or absence of pathologic lymph node after NAC. A recently published study demonstrated the potential utility of axillary ultrasound after NAC and before surgery to evaluate residual nodal disease in women presenting with node-positive breast cancer, showing that axillary ultrasound reduced the false-negative rate of SLNB from 12.6% to 9.8%.^31^ In addition, an independent cohort was included to validate our nomogram, and the independent validation indicated the good predictive power of the model.

During the study period, trastuzumab was not added to the NAC as a standard treatment in patients with HER2-positive tumors because it was not covered at the time by the national health insurance in Korea. Neoadjuvant trastuzumab therapy was approved in late 2013. Therefore, only 9.6% of the patients in our study received trastuzumab with their NAC, because they were participants in clinical trials (ie, NeoALTTO). As the number of patients receiving neoadjuvant trastuzumab has increased in recent years, the model construction might be modified to reflect the current use of neoadjuvant trastuzumab and the corresponding increased axillary pCR rate. Previous trials reported good response rates with taxane-containing regimens,^32^ and a taxane and no-anthracycline regimen was associated with a higher rate of axillary pCR in our study. Of 34 patients with a regimen based on taxane only, 13 patients were treated with concurrent trastuzumab, and 12 of those patients achieved axillary pCR. That result might be explained by the trastuzumab treatment in combination with taxane in HER2-positive tumors and not by the taxane-only regimen itself.

The biologic tumor subtype was associated with axillary pCR in this study, and HER2-enriched and triple-negative breast cancers were especially likely to achieve axillary pCR compared with luminal breast cancer. The rate of axillary pCR was 49.4% (38 of 77 patients) in the HER2-enriched tumors and 53.8% (50 of 93 patients) in the triple-negative tumors in our study. Our results are somewhat discordant with those of a previous study^33^ based on ACOSOG Z1071, which showed 64.7% and 49.4% rates of axillary pCR in HER2-enriched and triple-negative tumors, respectively. The markedly lower rate of axillary pCR in HER2-enriched tumors in our study might be related to the apparently lower rate of neoadjuvant trastuzumab treatment (9.6%) compared with that in the study based on ACOSOG Z1071 (88.9%). Although the biologic tumor subtype was associated with axillary pCR, we did not incorporate the biologic tumor subtype into the nomogram. Because the definition of biologic subtype varies somewhat among studies^34^ and the factors that determine the biologic subtype (ER, PR, and HER2 status) were incorporated in our nomogram separately, we chose to exclude the biologic subtype from the nomogram for simplicity.

The nomogram uses easily assessable clinicopathologic variables, allowing simple and rapid calculation of the probability of achieving axillary pCR. It can be used to help surgeons and patients making treatment decision and can also be utilized in clinical trials. The nomogram should be considered an ancillary tool, however, that provides measurable information but does not provide an actual clinical recommendation.

There are several limitations to our study. First, our nomogram was not validated in an external cohort. Although we validated it with an independent dataset, the validation data were extracted from the same institution that produced the training set. Second, as with any retrospective study, there is the issue of selection bias. Because the study included only patients who had cytologically confirmed nodal metastasis, patients who had false-negative fine-needle aspiration biopsy but received NAC were not included. Finally, the assessment of the clinical tumor and nodal responses to NAC using ultrasound alone might be somewhat subjective.^35,36^ Moreover, anatomical measurement alone might be insufficient to detect the tumor and nodal responses without considering functional and metabolic changes. Future efforts are needed to include more objective imaging tools like MRI^37^ and to reflect functional responses through changes in the maximum standardized uptake value (SUV_max_) on PET-CT.^38,39^

## CONCLUSION

Our study demonstrates a nomogram for calculating the probability of achieving axillary pCR in patients with cytologically proven node-positive breast cancer based on the known clinicopathologic features before and after NAC. The nomogram can help identify patients with a high probability of achieving axillary pCR who could be spared ALND, avoiding postoperative morbidity. To select such patients in actual clinical practice, further research on the accurate detection of residual tumor in the axilla must be explored in depth.
